# A cross‐sectional clinical study in women to investigate possible genotoxicity and hematological abnormalities related to the use of black cohosh botanical dietary supplements

**DOI:** 10.1002/em.22516

**Published:** 2022-11-28

**Authors:** Stephanie L. Smith‐Roe, Stavros Garantziotis, Rebecca L. Church, Jeffrey C. Bemis, Dorothea K. Torous, Kim G. Shepard, Cheryl A. Hobbs, Suramya Waidyanatha, Esra Mutlu, Keith R. Shockley, Grace E. Kissling, Sandra J. McBride, Guanhua Xie, Tim Cristy, Jessica Pierfelice, Kristine L. Witt

**Affiliations:** ^1^ Division of Translational Toxicology National Institute of Environmental Health Sciences Research Triangle Park North Carolina USA; ^2^ Clinical Research Branch, Division of Intramural Research National Institute of Environmental Health Sciences Research Triangle Park North Carolina USA; ^3^ Litron Laboratories Rochester New York USA; ^4^ Genetic and Molecular Toxicology Program Integrated Laboratory Systems, LLC (an Inotiv Company) Research Triangle Park North Carolina USA; ^5^ Biostatistics and Computational Biology Branch National Institute of Environmental Health Sciences Research Triangle Park North Carolina USA; ^6^ Social and Scientific Systems, Inc. A DLH Holdings Corp Durham North Carolina USA; ^7^ Battelle Columbus Ohio USA

**Keywords:** *Actaea racemosa* L., folate levels, herbal remedy, hormone replacement therapy, megaloblastic anemia, micronuclei

## Abstract

Black cohosh (BC; *Actaea racemosa* L.), a top‐selling botanical dietary supplement, is marketed to women primarily to ameliorate a variety of gynecological symptoms. Due to widespread usage, limited safety information, and sporadic reports of hepatotoxicity, the Division of the National Toxicology Program (DNTP) initially evaluated BC extract in female rats and mice. Following administration of up to 1000 mg/kg/day BC extract by gavage for 90 days, dose‐related increases in micronucleated peripheral blood erythrocytes were observed, along with a nonregenerative macrocytic anemia resembling megaloblastic anemia in humans. Because both micronuclei and megaloblastic anemia may signal disruption of folate metabolism, and inadequate folate levels in early pregnancy can adversely affect neurodevelopment, the DNTP conducted a pilot cross‐sectional study comparing erythrocyte micronucleus frequencies, folate and B12 levels, and a variety of hematological and clinical chemistry parameters between women who used BC and BC‐naïve women. Twenty‐three women were enrolled in the BC‐exposed group and 28 in the BC‐naïve group. Use of any brand of BC‐only supplement for at least 3 months was required for inclusion in the BC‐exposed group. Supplements were analyzed for chemical composition to allow cross‐product comparisons. All participants were healthy, with no known exposures (e.g., x‐rays, certain medications) that could influence study endpoints. Findings revealed no increased micronucleus frequencies and no hematological abnormalities in women who used BC supplements. Although reassuring, a larger, prospective study with fewer confounders (e.g., BC product diversity and duration of use) providing greater power to detect subtle effects would increase confidence in these findings.

## 1 INTRODUCTION

The National Toxicology Program, in response to concerns from the general public as well as the scientific community regarding the safety of botanical products, has been testing a variety of botanical materials for toxicological effects since 1998 (Abdel‐Rahman et al., 2011; National Toxicology Program [NTP], 2022b; Rider et al., 2018). One such botanical material, the dietary supplement black cohosh (BC), is marketed to women primarily to provide relief from vasomotor symptoms associated with menopause, but also to alleviate symptoms associated with other conditions such as premenstrual syndrome and menstrual cramps, as well as to induce labor (Gafner, 2016; National Center for Complementary and Integrative Health [NCCIH], 2020). Black cohosh is available in a variety of forms, including as an extract, root powder, tincture, or oral spray, and in combination with other botanicals. Black cohosh extract is made from the root of the plant, and over 100 constituents have been identified among various preparations (Nikolić et al., 2015; Qiu et al., 2014; Waidyanatha et al., 2022; Wang et al., 2011). Long used by Native Americans for a variety of medicinal purposes, BC was recorded in the first edition of the United States Pharmacopeia in 1820 (Mahady et al., 2008). The association between BC and relief from the symptoms of gynecological ailments arose in part from advertising by the Mrs. Lydia Pinkham Medicine Company, which incorporated BC extract as a constituent of a popular “vegetable compound remedy” sold to women in the late 1800 s to treat “female complaints.” Black cohosh has since become one of the most popular botanical dietary supplements sold in the United States (17th in sales in mainstream retail stores in 2020) and throughout the world (Smith et al., 2021).

Black cohosh was nominated to the DNTP by the National Cancer Institute and the National Institute of Environmental Health Sciences (NIEHS) due to widespread exposure, limited information on safety, and sporadic case reports of hepatotoxicity. Because BC is marketed as an alternative to hormone replacement therapy, the majority of clinical studies conducted with BC focused on the effectiveness of BC in alleviating discomfort associated with symptoms of menopause, rather than on toxicity; however, well‐controlled studies with exposures lasting from 3 to 12 months suggested that standardized BC extract formulations were no more effective than placebo treatment (Geller et al., 2009; Laakmann et al., 2012; Moore et al., 2017; NCCIH, 2020). A meta‐analysis that evaluated six randomized clinical trials with over 1000 subjects also concluded that the evidence for the ability of BC to alleviate symptoms of menopause was weak (Borrelli & Ernst, 2008). Regarding safety, no evidence of hepatotoxicity was observed in clinical studies that monitored this endpoint (Geller et al., 2009; Raus et al., 2006; van Breemen et al., 2010; Wuttke et al., 2006), while other measures of toxicity were rarely addressed. Thus, the available data suggested that benefits from BC use were minimal, while the potential for toxicity had not been thoroughly investigated.

In response to the nomination of BC, the DNTP initially conducted standard 90‐day toxicity studies in female Wistar Han rats and B6C3F1/N mice using a BC extract bearing a chromatographic profile similar to Remifemin® Menopause Tablets, a popular, standardized, BC product (Waidyanatha et al., 2022). Results showed that BC extract, administered by gavage at doses ranging up to 1000 mg/kg/day, was not hepatotoxic and did not modulate estrogenic signaling (Mercado‐Feliciano et al., 2012). However, significant, dose‐related increases in micronucleated red blood cells, indicative of induced chromosomal alterations, were observed in peripheral blood of both species at the end of the 90‐day studies. Furthermore, rats and mice developed a nonregenerative macrocytic anemia that resembled megaloblastic anemia in humans, which arises from disruption of the folate 1‐carbon metabolism pathway (Mercado‐Feliciano et al., 2012; Wickramasinghe, 2006). These two toxicological effects seen in rats and mice exposed to BC extract may have been related, as deficiency for folate or vitamin B12 (cobalamin, part of the folate metabolism pathway) has been shown to increase micronuclei (MN) in erythrocytes of humans (Everson et al., 1988; MacGregor et al., 1997), mice (LeBlanc et al., 2018; MacFarlane et al., 2015), and cultured human cells (Fenech, 1999).

The increase in MN observed in the DNTP animal studies and the possible implications for dysregulation of folate metabolism raised serious concerns, as elevated frequencies of MN and decreased levels of folate following an exposure indicate a potential for a substance to cause cancer, birth defects, or infertility (Duthie, 1999; MRC Vitamin Study Research Group, 1991). These observations prompted the DNTP to initiate a 2‐year rodent bioassay to assess the potential for BC extract to induce tumors in B6C3F1/N mice and Harlan Sprague Dawley rats (presently on test). In this ongoing study, induction of MN was observed in interim blood samples obtained from the mice at 3‐month and 12‐month intervals, confirming the observations in the original 90‐day toxicity study (NTP, 2022a). In an additional study by the DNTP designed to further explore the hematological effects of BC extract, female B6C3F1/N mice were exposed to 1000 mg/kg/day BC extract for 3 months and increases in Howell‐Jolly bodies (MN) were observed along with several lines of hematological evidence consistent with a functional vitamin B12 deficiency (Cora et al., 2017).

Translating animal findings to human exposure scenarios to understand potential health risks associated with the exposures, requires a consideration of the dosing differentials. In the case of BC, the recommended doses for a variety of different extracts currently on the market range from 40 to 250 mg/day, with some as high as 600 mg/day. Using allometric scaling based on surface area (Reagan‐Shaw et al., 2008), these recommended human doses are approximately 40‐ to 2‐fold lower, respectively, than the lowest dose (250 mg/kg/day) of the DNTP BC extract that induced significant increases in MN in female mice. Thus, the human doses, although lower than the animal doses, may fall within the standard “10‐fold” safety margin in dosing differential between animals and humans. To assess the translatability of the observed toxicological responses in animals, the DNTP, in collaboration with the NIEHS Clinical Research Unit (CRU), designed and conducted a cross‐sectional clinical study to investigate whether women who regularly used BC supplements showed increased frequencies of MN and evidence of dysregulation of folate metabolism compared to BC‐naïve women. The concerns that prompted this study were threefold: BC is marketed to women of child‐bearing age as well as to post‐menopausal women, use of BC may extend for years, and women may consume more than the recommended amount of BC per day. Several standard hematological and clinical chemistry parameters with well‐characterized methods of analysis were also evaluated in the study participants. In addition, chemical profiles of the supplements used by participants were generated to allow comparisons in chemical composition among the different products and to compare each of the products to the DNTP BC extract (Waidyanatha et al., 2022) that induced MN in the rodent studies (Mercado‐Feliciano et al., 2012).

## 2 MATERIALS AND METHODS

### 2.1 Participant recruitment and selection

All participant recruiting and interviewing, as well as blood draws were conducted at the NIEHS CRU in accordance with protocols approved by the NIEHS Institutional Review Board (IRB #10‐E‐0063). All participants gave informed consent before donating blood samples. All study participants and the blood samples they provided were assigned coded identification numbers so that testing laboratories and DNTP staff were blinded to their identity.

Two groups (BC‐exposed and BC‐naïve) of non‐pregnant females, 18–70 years of age, were recruited by open advertisement (e.g., local newsletters, flyers posted on bulletin boards and at local retailers) from the Research Triangle area of central North Carolina as well as by invitation from the NIEHS Personalized Environment and Genes Study (PEGS) (https://joinastudy.niehs.nih.gov/studies/pegs/index.htm) from May 2014 through September 2017. BC‐exposed participants were selected based on ≥3 months use of a commercial BC product purchased from a local retail store (e.g., grocery store, drug store). Users of combination botanical products that included BC were excluded from the study. BC‐naïve women were recruited from other ongoing studies at the NIEHS Clinical Research Unit.

All participants were self‐reported as healthy, with no chronic diseases (e.g., liver disease, myelodysplastic syndrome), and no previous cancers (surrogate for chemotherapy treatment with genotoxic drugs). Participants were prescreened by telephone for use of medications or other botanical products that might influence folate or B12 levels (e.g., metformin, sulfasalazine), or potentially induce chromosomal alterations in the form of chromosome loss (aneuploidy) or breakage (clastogenicity). Exposures to antiretroviral medications or radioactive iodine 131 were exclusion factors. In addition, participants were excluded from the study if they had received diagnostic x‐rays, other than dental x‐rays, in the past 3 months.

Participants provided a complete medical history including current prescription and over‐the‐counter medications, dietary supplements (including use of multivitamins and vitamin B12), alcohol use, cigarette use, medical conditions, and past medical procedures. Information on the identity of the BC product used (brand name, lot number) and duration of use was collected from each participant, and participants were asked if they were willing to provide a sample of the product for chemical analysis.

### 2.2 Black cohosh supplement and blood sample collection

Successfully prescreened participants were scheduled for an appointment at the NIEHS CRU. During this appointment, additional screening was conducted. Qualified participants then provided informed consent and blood samples were collected. Black cohosh supplements provided by participants at the time of their clinic visit were stored individually in a 10 ml cryovial (Denville Scientific, item # V8910‐A) at 4°C and shipped in batches to Battelle (Columbus, OH) for chemical analysis. If available, supplement packaging was photographed to document manufacturer, lot number, ingredients, and recommended daily dose.

Blood samples were drawn by trained phlebotomists using a metal 21‐gauge butterfly needle (Cardinal, cat. # B3036‐21), attached to a vacutainer needle holder (Cardinal, cat. # 22‐289‐953). Blood was collected into four 3 ml lavender top K_2_EDTA vacutainer blood tubes (Fisher Scientific, cat. # 02‐683‐99B), and one Greiner vacutainer blood tube containing microscopic silica particles with separation gel (Fisher Scientific, cat. #22‐040‐536). One lavender top tube was packaged immediately, refrigerated, and transferred the same day to Integrated Laboratory Systems, LLC (ILS, an Inotiv Company; Research Triangle Park, NC) for processing prior to assessment of erythrocyte MN frequencies. The second lavender top tube was packaged according to Quest Diagnostics (Cary, NC) guidelines and shipped for complete blood count (CBC) analysis. The third lavender top tube was packaged on cold packs and shipped to the NIH Clinical Center (Bethesda, MD) for reticulocyte measurements. The fourth lavender top tube was centrifuged to isolate plasma, and the plasma was then dispensed into a 4.5 ml cryotube, frozen, and sent to the NIH Clinical Center on dry ice for measuring homocysteine levels. The Greiner tube was centrifuged to isolate serum and was sent on cold packs to the NIH Clinical Center for measuring vitamin B12 and folate levels. All blood samples were coded at the NIEHS CRU before distribution for analysis.

### 2.3 Clinical chemistry

Clinical chemistry analyses were conducted using standard well‐characterized methods at the NIH Clinical Center, a Certified Analytics Professional (CAP) laboratory with Clinical Laboratory Improvement Amendments (CLIA) certification. Serum levels of vitamin B12 and folic acid were determined using the IMMULITE 2000 chemiluminescent diagnostic assays for vitamin B12 (Siemens, cat. # L2KVB2) or folic acid (Seimens, cat. # L2KFO2) and analyzed using a Siemens Healthcare Diagnostics IMMULITE 2000 XPi imaging system. Plasma levels of homocysteine were determined using the Homocysteine Enzymatic Assay (Roche, cat. # 05385415) and analyzed using a Roche cobas® 6000 Analyzer.

### 2.4 Micronucleus assay

The collection and shipping of human blood samples was performed according to instructions supplied with the BASIC Human In Vivo MicroFlow kit (Litron Laboratories, Rochester NY) (Dertinger et al., 2011). Briefly, within 3 h of receipt at ILS, whole blood samples collected in K_2_EDTA vacutainer tubes were diluted in anticoagulant, fixed in ultra‐cold methanol, and stored in a −80°C freezer. Within 3 to 5 days of fixation, samples were transferred to kit‐supplied Long‐Term Storage Solution (LTSS) and stored in a −80°C freezer for at least an additional 2–3 days. Samples were shipped in batches overnight in temperature‐controlled packaging to Litron Laboratories (Rochester, NY). Upon receipt, samples were stored in a −85 °C freezer until further processing. On the day of analysis, samples were washed out of LTSS and labeled with α‐CD71‐FITC (reticulocytes) and α‐CD61‐PE (platelets). The cells were then incubated with α‐FITC microbeads (Miltenyi Biotech, Bergish‐Gladbach, Germany), a pre‐column sample was obtained, and the remainder of each sample was eluted through magnetic columns to enrich for the highly CD71‐expressing reticulocyte (RET) population (most immature population of erythrocytes). Both pre‐ and post‐column samples were labeled with a nucleic acid dye prior to analysis on a Becton Dickinson FACSCalibur™ flow cytometer running Cell Quest™ Pro (v5.2) software. The gating strategy enabled enumeration of the following populations: RET, micronucleated RET (MN‐RET), normochromatic erythrocytes (NCE), and micronucleated NCE (MN‐NCE). Pre‐column samples provided %RET and %MN‐NCE and post‐column data were used to calculate %MN‐RET. It should be noted that although %MN‐NCE data were collected concurrently with the other endpoints in the micronucleus assay, they did not serve as a measure of chromosomal damage because in humans, the healthy spleen removes micronucleated erythrocytes from circulation soon after they emerge from the bone marrow; thus, in humans, the appropriate population for assessing micronucleated erythrocyte frequencies is the immature reticulocyte population (Witt et al., 2007).

### 2.5 Chemical analysis of black cohosh supplements

Samples of supplements provided by study participants were analyzed by Battelle (Columbus, OH). Standards for quantitation of constituents of cohosh materials (Table S1), cohosh reference materials (Table S2), and all other reagents were procured from commercial sources. For sample preparation, deionized water (ASTM Type I) was used. Stock solutions of individual standards were prepared in 80:20:0.1 methanol:water:formic acid (extraction solvent) at target concentrations of ~2000 μg/ml. These individual standards were used to prepare combined standard solutions at 300 μg/ml. The combined standard solution was subsequently diluted with the same extraction solvent to generate calibration standards in duplicate in the target range of 18–150 μg/ml with respect to each analyte.

Cohosh reference materials and the DNTP BC extract were prepared by extracting ~200 mg of each sample with 5 ml of extraction solvent to generate approximately 40 mg/ml with respect to black cohosh. For some study participant samples the information on the amount of BC per unit of supplement was not available. Therefore, for all study participant samples, one unit of supplement (e.g., one tablet) was used for analysis. Each sample was weighed and then prepared for analysis as follows. Tablets (9 supplements) were ground using a mortar and pestle. The mortar and pestle were rinsed with 1 ml of extraction solvent twice and combined with the sample. Three milliliters of extraction solvent were then added to bring the final volume to 5 ml. Capsules (9 supplements) were opened, and contents were extracted with 5 ml of extraction solvent. The single softgel capsule was combined with 1 ml of deionized water and the tube was heated in a 40°C water bath. Methanol (2 ml) was added to aid the complete dissolution of the capsule and the final volume was brought to 5 ml by the addition of 2 ml of methanol.

All samples were vortexed for ~30 s and sonicated for 30 min with intermittent vortex mixing every 10 min. The samples were then rotated end‐over‐end at 70 rpm overnight and centrifuged at 3000 rpm for 5 min. The supernatant was analyzed by high performance liquid chromatography (HPLC) with charged aerosol detection (CAD) as described below. Solvent and extraction blanks were also prepared for analysis.

The high‐performance liquid chromatograph used was an Agilent 1100 (Santa Clara, CA) coupled to a Dionex Corona Veo (Thermo Fisher Scientific, Waltham, MA) charged aerosol detector. A Phenomenex (Torrance, CA) Aqua C18 column (250 × 4.6 mm, 5 μm) was used for analyte separation. Mobile phases A (1% aqueous formic acid) and B (acetonitrile) were used at 1 ml/min and with a linear gradient (%B): 5–15, in 15 min with a 5 min hold, 15–30 in 15 min, 30–40 in 15 min, 40–50 in 45 min and 50–95 in 5 min.

A linear regression with 1/*x* weighting was used to relate the peak area response to the concentration of calibration standards. Concentration of each marker was calculated using the peak area response and the regression equation. Standard curves were linear for all analytes (Table S3). Limit of detection (LOD) was estimated as the 3*X* the standard deviation of the lowest standard and is given in Table S3 for individual analytes. Analyte concentrations were reported as μg/g of tablet or capsule weight. All values above the limit of detection (LOD) of the analytical method were reported.

The chromatograms for the samples were imported into SpecAlign software v2.4.1 (University of Oxford, England) where the peaks were aligned, the baselines adjusted to the same level, and the data binned to reduce the data rate from 25 points per second to 5 points per second. This was done to remove minor instrument performance shifts over the course of the run set and to smooth the noise. The processed chromatograms were then imported as time (*X*) and detector response (*Y*) data into the Eigenvector Research Solo version 8.5.1 chemometrics software (Manson, WA). A hierarchical cluster analysis (HCA) using Ward's Method was used to create the dendrogram showing clusters of similarity.

### 2.6 Statistical analyses

Prestudy power calculations were based on the micronucleus and folate/B12 level endpoints, which were the main endpoints of interest in this study, and included the initial assumption that all BC‐exposed women would be using the same brand of supplement and that each BC‐exposed woman would have an age and race matched control. For the folate and B12 measures, enrolling 18 women per group (black cohosh‐exposed and black cohosh‐naïve) would provide 80% power to detect a 1.5‐fold difference between the two groups. Increasing group size to 24 participants would provide 90% power to detect a 1.5‐fold difference. The required sample size for the micronucleus endpoint, based on similar studies conducted with Diversity Outbred mice that mimic the genetic diversity in the human population, was the same as for the folate/B12 endpoints. Thus, based on these initial calculations, this study aimed to enroll 24 women in each of the two study groups.

For all statistical analyses, the BC‐exposed group was compared with the BC‐naïve group. Endpoints analyzed included %MN‐RET and %RET, along with standard clinical measures including reticulocyte absolute, reticulocyte hemoglobin, immature platelet fraction, immature reticulocyte fraction, homocysteine, serum folate, serum vitamin B12, white blood cell count, red blood cell count, hemoglobin, hematocrit, mean corpuscular volume (MCV), mean corpuscular hemoglobin (MCH), mean corpuscular hemoglobin concentration (MCHC), red cell distribution width (RDW), mean platelet count, mean platelet volume (MPV) (Table S4). Comparisons between exposed and control groups were performed using the Mann–Whitney *U* test, with the statistical significance defined as *p* < .05.

## 3 RESULTS

### 3.1 Study participants and supplement use

Recruitment to this study continued for a period of 3 years in an attempt to achieve the group sizes (~24 participants per group) recommended by prestudy power calculations. By the end of the 3‐year recruitment period, we were able to enroll 51 women in the study, 28 in the BC‐naïve group and 23 in the BC‐exposed group. Participant demographics are listed in Table 1. The mean age of the BC‐naïve group was 48.4 ± 1.9 (*SE*) (*n* = 27, age was not recorded for one participant), and for the BC‐exposed group, mean age was 52.7 ± 1.5 (*SE*). Four BC‐naïve and two BC‐exposed women were smokers. Smoking status was not recorded for 1 BC‐naïve woman.

**TABLE 1:** Participant demographics

Control		BC supplement	
Age (years)	Race	Age (years)	Race
25	African American	24	African American
32	White	43	African American
34	African American	49	White
34	African American	50	White
36	African American	51	White
39	African American	51	White
42	African American	53	White
44	African American	53	White
46	White	53	White
47	White	53	African American
47	African American	54	White
49	African American	54	White
50	White	54	African American
51	White	55	White
51	White	55	White
51	White	56	African American
51	African American	57	White
52	White	57	White
54	White	58	African American
54	White	58	Multiple
56	African American	58	African American
57	White	58	White
57	African American	59	White
59	White		
62	White		
62	Asian		
64	White		
(Missing^a^)	(Missing^a^)		
Mean age ± standard error			
48.4 ± 1.9		52.7 ± 1.5	

^a^ Age and race were missing for one control participant.

The BC supplements used by women in the BC‐exposed group were highly variable and included pills, capsules, root powders, and one liquid formulation. For 9 of the BC products, dosages as per label recommendations ranged from 40 to 120 mg/day, and for 5 root powder formulations, dosages ranged from 50 to 1620 mg/day (Table 2). For 9 products, dosage information was not provided, and for 7 of those, no information on whether the BC product was an extract or a root powder was available (Table 2). The duration of BC supplement use for participants in our study ranged from 3 months, the minimum requirement for inclusion in the study, to approximately 16 years (Table 2).

**TABLE 2:** Black cohosh dietary supplements taken by study participants and reported duration of use

Manufacturer	Lot number^a^	Form of supplement	Extract/pill (mg)	Root/pill (mg)	Recommended daily dosage	Duration of use
Supplements analyzed for chemical markers present in cohosh extracts and root powders						
General Nutrition Centers	3966F 01970	Capsule	40		1 capsule	5 years
Nature's Answer	18126	Capsule		50	1 capsule	3 months
Nature's Way	20038603	Capsule		540	3 capsules	1.5 years
Radiance	429900‐01	Softgel capsule	40		NA	>1 year
Remifemin^1^	437401	Tablet	20		2 tablets	1.75 years
Remifemin	541061	Tablet	NA^b^	NA	NA	6–12 months
Remifemin	552731	Tablet	NA	NA	NA	6 years
Spring Valley	5AN1300	Tablet	40		2 tablets	7 years
Spring Valley	5BN1561	Tablet	40		2 tablets	3–6 months
Spring Valley	5JN1907	Tablet	40		2 tablets	2.5 years
Spring Valley	27878 2G F1	Tablet	40	NA	NA	>1 year
Spring Valley	6EN2090	Tablet	NA	NA	NA	6–12 months
Spring Valley	214490250	Capsule	40	135	3 capsules	> 1 year
Spring Valley	NA	Capsule	40	135	NA	15–16 years
Swanson	SW1344 B21408	Capsule		540	1 capsule	15 years
Up & Up	5CN1255	Tablet	40		2 tablets	3–6 months
The Vitamin Shoppe	131171	Capsule	40		NA	6–12 months
Weaver Street	0930X 100 419133	Capsule	NA	NA	NA	3 years
Whole Foods	410039	Capsule		100	1 to 2 capsules	3 months
Supplements not provided for chemical analysis						
Gaia	1280011003	Liquid	NA	NA	40 drops	6 years
Remifemin	20016889	NA	NA	NA	NA	2 years
Spring Valley	300005	Tablet	40		2 tablets	6–12 months
Up & Up	6KN1354	Tablet	40		2 tablets	2 years

*Note*: “Remifemin Tablet 1” in Figure 1.

^a^ Different lots from the same brand and type of pill are in the same order in Figure 1; for example, the Remifemin tablet with lot number 437401 is “Remifemin Tablet 1” in Figure 1.

^b^ Not available.

### 3.2 Analysis of micronucleated peripheral blood cells

Flow cytometric analysis of blood samples to determine %MN‐RET and %RET showed no significant differences in mean values between the two groups of participants (Table 3), and for both groups of participants, mean values were within previously reported control ranges for the analytical laboratory (Torous et al., 2020; Witt et al., 2007). Individual data were available for 22 participants in the BC‐naïve group and 23 in the BC‐exposed group; the values are shown in Table S5. Values for %MN‐NCE were collected concomitantly during the analysis procedure but were not used as a measure of chromosomal alterations because in humans, the healthy spleen quickly and efficiently removes damaged red blood cells from circulation (Witt et al., 2007), and there was no evidence of splenic malfunction in participants in our study.

**TABLE 3:** Micronucleus assay endpoints

Endpoint^a^	Group	*n* ^b^	Mean ± *SE* ^c^ ^,^ ^d^	25%	Median	75%	*p* value
%MN‐RET	Control	22	0.228 ± 0.04	0.140	0.180	0.230	.132
	Supplement	23	0.290 ± 0.04	0.170	0.230	0.340	
%RET	Control	22	0.144 ± 0.02	0.063	0.102	0.183	.073
	Supplement	23	0.090 ± 0.01	0.037	0.083	0.132	

^a^ %MN‐RET, percent micronucleated reticulocytes; %RET, percentage of reticulocytes among total circulating erythrocytes.

^b^ Data not available for every participant in the Control group.

^**c**^ Mean value for %MN‐RET reported by Torous et al., 2020 for 338 males and females ranging in age from birth (cord blood) to 73 years was 0.15 ± 0.10%; range, 0.01–0.79%.

^**d**^ Range for %RET reported by Torous et al., 2020 was generally 0.1–0.3%.

### 3.3 Analysis of folate, vitamin B12, and homocysteine

No significant differences were observed between the two study groups in levels of serum vitamin B12 or homocysteine (Table 4). The mean values for both groups were within normal ranges for serum vitamin B12 and homocysteine. The mean serum folate level exceeded the upper bound of the reference range of 3.0–17.0 ng/ml for the supplement group (21.86 ± 2.2 ng/ml) and was significantly elevated over the mean value for the control group (*p* = .036). Serum folate levels are closely linked to recent dietary intake and may not reflect long‐term serum folate status (https://ods.od.nih.gov/factsheets/Folate-HealthProfessional/). The majority of women in our study who were using BC supplements also used multivitamin and vitamin B12 supplements; fewer participants in the BC‐naïve group reported vitamin use (Supporting Information, Figure S1). The use of vitamin supplements may have compensated for any subtle effects of BC use on folate, B12, or homocysteine levels.

**TABLE 4:** Levels of serum folate, serum vitamin B12, and homocysteine

Endpoint^a^	Group	*n* ^b^	Mean ± *SE*	25%	Median	75%	*p* value
Serum folate (ng/ml)	Control	25	16.68 ± 2.7	8.2	12.9	19.5	.036^c^
	Supplement	23	21.86 ± 2.2	13.7	22.0	28.7	
Serum vitamin B12 (pg/ml)	Control	25	590.4 ± 66	386.0	475.0	678.0	.200
	Supplement	23	749.8 ± 90	384.0	690.0	998.0	
Homocysteine (μmol/L)	Control	22	13.18 ± 2.6	8.0	9.5	13.0	.521
	Supplement	19	10.00 ± 0.7	8.0	9.0	11.0	

^a^ Historical reference ranges for NIH Clinical Center: serum folate, 3.0–17.0 ng/ml; serum vitamin B12, 93–982 pg/ml; homocysteine, 0–13 μmol/L.

^b^ Number of participants in each group for which data were available for each of the 3 endpoints.

^c^ Significant at *p* < 0.05.

### 3.4 Analysis of hematological endpoints

No significant differences were observed between the BC‐exposed and BC‐naïve groups for the following endpoints: %RET, absolute reticulocytes, reticulocyte hemoglobin, immature platelet fraction, immature reticulocyte fraction, white blood cell count, red blood cell count, hemoglobin, hematocrit, MCV, MCH, platelet count, and MPV. The mean values for both groups were within normal ranges. A significant decrease in red cell distribution width (RDW) was observed for supplement users (*p* < .008) (Table S4). However, the mean values for both groups were within the normal range (12.2%–16.1% for women).

### 3.5 Chemical analysis of black cohosh supplements

BC supplements provided by the study participants were analyzed for the 14 constituents listed in Table S1. Constituents reported in the literature to be present in BC materials were not uniformly present in the supplements (Supporting Information, File 1). Isoferrulic acid, cimiracemoside C, and actein/27‐deoxyactein (these two constituents co‐eluted) were more frequently detected across all the supplements compared to other constituents. No single constituent was detected in every supplement.

**FIGURE 1:**
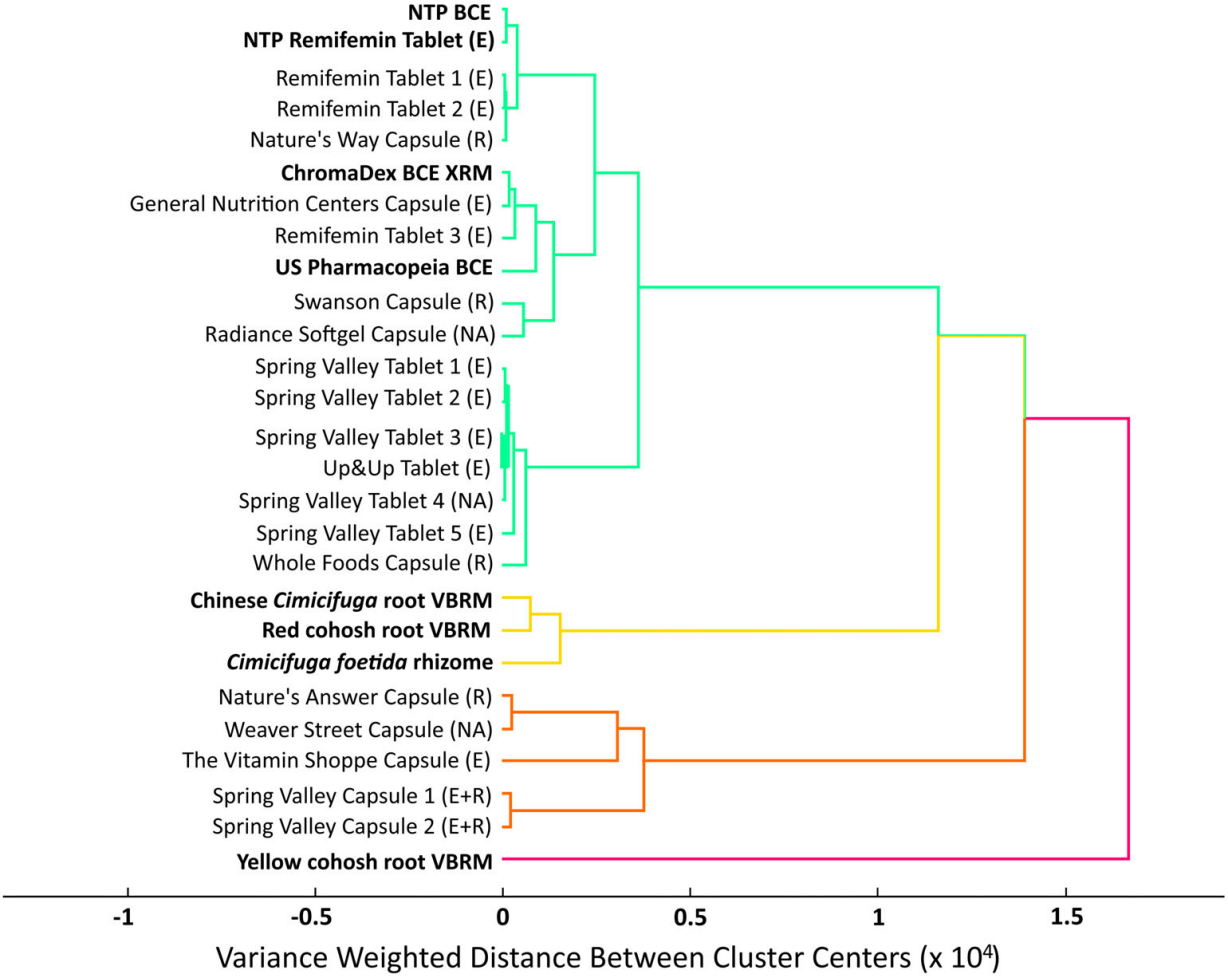
Dendrogram indicating the relatedness between the sample of BCE found to induce micronuclei in 90‐day rat and mouse studies conducted by the NTP (“NTP BCE”), BCE reference materials (BC XRM from ChromaDex, BCE from US Pharmacopeia), a Remifemin tablet characterized by NTP prior to this study (NTP Remifemin Tablet), other species of cohosh (bold text), and supplements taken by participants based on analysis of 14 chemical biomarkers (see Table S1). E, extract; R, root powder; NA, not available

Chromatographic profiles of samples were evaluated along with the BC extract used for DNTP research (“NTP BCE”), a Remifemin tablet previously characterized by the NTP, and standard reference materials for other cohosh materials (Table S2) using hierarchical clustering. The corresponding data are shown in Figure 1. Samples of Remifemin® supplements provided by participants in this study clustered together with the NTP BCE as well as to the BCE reference material from ChromaDex (BC XRM) and the BCE procured from the US Pharmacopeia (Figure 1). Overall, 18 of the samples provided by participants grouped together and were considered chemically similar to black cohosh preparations in general; these 18 samples showed a clear separation from other species of cohosh. The remaining 5 supplements, which showed distinct differences in composition from the other 18 samples, may have been adulterated with other cohosh species.

## 4 DISCUSSION

Results from this study suggest that risks of chromosomal damage, as indicated by the frequency of micronucleated reticulocytes, and serum folate levels in women who regularly use BC supplements, may be low. Although data were not available for every participant, no significant difference was seen between BC‐exposed and BC‐naïve women in the frequency of micronucleated reticulocytes. Interestingly, the mean serum folate level for participants in the BC‐exposed group was higher than the upper bound of the normal reference range and was significantly higher than the mean value for the BC‐naïve group (p < 0.036). It is possible that serum folate levels may have been influenced by the regular use of multivitamins and/or vitamin B12 reported by most participants in the BC‐exposed group but not in the BC‐naïve group. Since dietary fortification with folate was implemented in the U.S. in 1998, studies have suggested that the incidence of high blood folate levels has increased within the U.S. population (Smith et al., 2008), and serum folate measurements reflect recent dietary intake. Erythrocyte folate levels provide a more stable measure of long‐term folate status, but these were not measured in our study (https://ods.od.nih.gov/factsheets/Folate-HealthProfessional/). The sources of variation in this study, particularly product variability and duration of exposure to BC, leave open the question of undetected subtle effects associated with long‐term chronic use of BC products.

In our study, we had 23 participants in the BC‐exposed group for which we were able to analyze most endpoints, and 28 women were included in the control (BC‐naïve) group. Although target group sizes (~24 participants) derived from the pre‐study power calculations were achieved, the power to detect significant differences in the endpoints between the two groups of women in this cross‐sectional study was limited by confounding factors such as the variety of products that were used by the women in the BC‐exposed group, the duration of product use, incomplete data for certain endpoints, and variable use of multivitamins among women in both study groups. The degree of lifestyle and product variation between the two groups, and among the women who used BC products in our study might have been reduced, had we conducted a prospective study and supplied a single BC supplement for use by all women in the BC‐exposed group, or if we had been able to recruit women to the exposure group who were all using the same BC product. In addition, in a prospective design, each study participant in the BC‐exposed group would have been able to serve as their own control by providing blood samples before beginning BC use and then again after at least 3 months of daily product use. Aware of the variety of BC supplement products on the market, the study protocol initially limited enrollment to women who were using a Remifemin BC product, due to the anticipated similarity in constituent profile to the NTP BC extract (and supported by Figure 1) and to ensure standardization of product among the participants. However, few women using that brand of supplement responded to the study advertisement and therefore, after several months of recruiting with little success, we broadened the enrollment criteria to include the use of any single (i.e., no additional botanicals included) BC product. A further challenge to recruiting was the tendency for users of BC to use multiple botanical/herbal products. In addition, our limited advertising budget precluded use of newspaper advertisements that would have reached a much broader population of women than were reached through local outreach efforts (e.g., posted flyers in stores). Thus, multiple factors contributed to the diversity in participant characteristics and product use.

In addition to the factors described above regarding the limitations to data interpretation in this study, the cross‐sectional study design itself poses a challenge to data interpretation, as cross‐sectional studies cannot differentiate between cause and effect; they can only identify differences between two or more study groups. As indicated in the methods description, this study was by design a “pilot” study and the intent was to determine if there were significant identifiable differences between women who used BC products and women who did not to support conducting a larger, prospective (i.e., cohort) study.

The limitation posed by the diversity of products used by participants in this study derives from a basic challenge in studying the toxicological effects of botanical materials: chemical composition of the preparations can vary considerably due to, e.g., differences in growing conditions, parts of the plant extracted, solvents used for extraction, manufacturing processes, and adulteration (Ryan et al., 2019; Shipkowski et al., 2018; Waidyanatha et al., 2022). Variable composition can lead to variable biological activity. Thus, to investigate whether different species of cohoshes and different formulations of BCE possessed similar abilities to induce MN, the DNTP recently conducted in vitro studies in human TK6 lymphoblastoid cells with the DNTP BC extract used in animal studies and several additional cohosh extracts and root powders as well as different species of cohoshes obtained as standard reference materials (Smith‐Roe et al., 2018). Results confirmed that the ability of the DNTP BC extract to induce MN in rodents in vivo was also observed in human cells in vitro, and importantly, this induction of MN was a characteristic of cohoshes in general (Smith‐Roe et al., 2018). MN may be formed via two basic mechanisms: whole chromosome loss (aneugenicity), or failure of an acentric, broken chromosome (clastogenicity) to migrate to a spindle pole during mitosis. The DNTP BC extract was shown to induce MN through destabilization of microtubules, resulting in whole chromosome loss during mitosis (Bernacki et al., 2019). Additional studies have provided some evidence that the DNTP BC extract may have transient DNA‐damaging effects as well (Seo et al., 2021). Bio‐fractionation studies are currently underway by the DNTP aimed at identifying the constituents in the DNTP BC extract that are responsible for the observed genotoxicity.

A significant decrease in RDW was observed for supplement users compared with the controls (p < 0.008), while the mean value for both groups was within the normal range for this endpoint (Table S4). Furthermore, although changes in RDW may signal conditions such as anemia, kidney disease, or diabetes, RDW is only one endpoint measured in a comprehensive complete blood count (CBC), and RDW data are evaluated in context. No values out of range were seen among the other CBC endpoints measured in our study and therefore, the lower mean RDW value in the BC‐exposed group likely does not signal an adverse health effect.

Although BC supplements are primarily used by menopausal and post‐menopausal women, certain brands of BC supplements are marketed specifically to women of child‐bearing age to relieve painful and uncomfortable menstrual symptoms. In younger women, the potential for BC to disrupt the folate metabolism pathway, resulting in reduced serum folate and elevating the risk for neurodevelopmental abnormalities during early pregnancy was a major concern and was the primary reason for initiating this study. As older women may tend to have decreased levels of vitamin B12, which can lead to irreversible neurological symptoms, the possibility that BC supplements might disrupt folate metabolism in older women was also a concern, as such an effect might further depress vitamin B12 levels. Our results revealed no deficiencies in B12 levels in any of the women in our study. It should be noted that high levels of blood folate might mask vitamin B12 deficiencies, and a few participants in our study did exhibit high blood folate levels. In both age groups, we were concerned about the possibility that BC might induce chromosomal damage in bone marrow cells, but in contrast to the observations in rodents (Mercado‐Feliciano et al., 2012) and in human cells in vitro (Smith‐Roe et al., 2018), we saw no evidence of this process in the women in our study, regardless of age.

Given the observations of BCE‐induced MN induction in rodents and in cultured human cells, in contrast to the negative results seen in the women in our study, we compared the doses of BCE administered to rodents and the standard recommended doses for the products used by participants in our study. The product‐recommended daily doses for the black cohosh extracts used by women in our study ranged from 40–120 mg/day, with 40 mg/day the most commonly recommended dose. The BCE dose level of 250 mg/kg/day that induced a significant increase in MN in mice in the 3‐month studies conducted by the NTP (Mercado‐Feliciano et al., 2012) was approximately 30– to 40–fold higher than the 40 mg/day dose recommended for women who use popular products such as Remifemin. A pharmacokinetic study was conducted in female volunteers using an authenticated BC extract preparation (doses up to 128 mg/day) and monitoring one of the few identified BC extract constituents, 23‐epi‐26‐deoxyactein, representing just ~2% of the total extract, for 24 h following oral administration (van Breemen et al., 2010). Results revealed extremely low levels of the parent compound in urine, and no metabolites in urine or serum. This study also measured liver enzymes during the 24‐h dosing period and observed no changes, indicating an absence of acute hepatotoxicity from BCE ingestion. Furthermore, no evidence of hepatotoxicity was seen in a 12‐month prospective double‐blind clinical trial with a standardized black cohosh product (Geller et al., 2009). In addition, 23‐epi‐26‐deoxyactein was shown to be degraded during incubation in simulated stomach acid, suggesting that it may be degraded in the human stomach following ingestion of black cohosh (van Breemen et al., 2010). Although intriguing, we cannot extrapolate these findings from a single constituent representing just ~2% of the total extract. Approximately 94% of the constituents of black cohosh remain unidentified and the compounds responsible for the observed genotoxicity in the DNTP studies have not been identified. Detailed characterization of the composition of the NTP BCE using targeted and non‐targeted chemical analysis confirmed the suitability of the sample (authentic, non‐adulterated, close resemblance to the popular product Remifemin) for use in pre‐clinical studies (Waidyanatha et al., 2022). Furthermore, findings from the DNTP fractionation studies, aimed at identifying the specific genotoxic constituents(s) of BC extract, may support refined ADME studies for BC extract in humans and allow a more accurate interpretation of health hazard, if any, associated with the recommended doses of BC products.

Although the results of this cross‐sectional study were largely negative (i.e., no differences signaling adverse health status were observed in measured biomarkers between women who used BC products and BC‐naïve women), the limitations noted above in our study design leave open the possibility that subtle but significant biological effects may not have been detected. Therefore, we suggest that additional studies employing prospective (i.e., “cohort”) designs, including measures similar to those evaluated in our study, and enrolling a well‐matched control group may provide a more definitive assessment of the safety of BC products, when these are used in accordance with product label recommendations.

## STATEMENT OF AUTHOR CONTRIBUTIONS

Conception and design of the study (KLW, GEK, SG, RLC, JCB, CAH); design of experiments and acquisition of the data (KLW, SW, EM, CAH, KGS, JCB, DKT, SG, RLC, TC, JP); analysis and interpretation of the data (SJM, GX, SW, EM, KLW, DKT, KRS, GEK, SLS); drafting of the article or revising it critically for intellectual content (SLS, KLW, SW, SJM, GEK, CAH, KRS).

## CONFLICT OF INTEREST STATEMENT

DKT and JCB are employed by Litron Laboratories, a company that sells MicroFlow reagent kits and offers testing services based on this assay. Litron has also received funding from the National Institute of Environmental Health Sciences in the form of SBIR grants for the development of the MicroFlow assay.

## Supporting information


**Table S1** Standards used to evaluate black cohosh dietary supplements taken by study participants.

**Table S2** Cohosh materials compared to black cohosh dietary supplements taken by study participants.

**Table S3** Analysis method parameters for quantitation of black cohosh materials. Coefficients of determination for standards: limit of detection (LOD), lower limit of quantitation (LLOQ), and upper limit of quantitation (ULOQ)

**Table S4** Hematological endpoints.

**Table S5** Participant micronucleus values.

**Figure S1** (a) Serum folate levels in study participants and multivitamin use: Low folate, < 3.0 ng/ml; normal folate, 3.0–17.0 ng/ml, high folate, > 17.0 ng/ml. (b) Serum vitamin B12 levels in study participants based on whether they were taking a multivitamin and/or vitamin B12: low B12, < 93 pg/ml; normal B12, 93–982 pg/ml; high B12, > 982 pg/ml. Not all participants had a measurement for serum folate or serum vitamin B12 levels, or information on use of multivitamins.

**Appendix S1:** Supporting Information.
