# Supplementary material for: A cross‐sectional clinical study in women to investigate possible genotoxicity and hematological abnormalities related to the use of black cohosh botanical dietary supplements
Source: Environ Mol Mutagen. 2022 Nov 28;63(8-9):389–99. doi: 10.1002/em.22516 (PMC10018809; doi:10.1002/em.22516)
Supplement: Supplementary file 1 — Table S1 Standards used to evaluate black cohosh dietary supplements taken by study participants. [file EM-63-389-s005.docx]

**SUPPORTING INFORMATION TABLES**

**TABLE SI** Standards used to evaluate black cohosh dietary supplements taken by

study participants

|  |  |  |
| --- | --- | --- |
| **Standard** | **CASRN** | **Supplier** |
|  |  |  |
|  |  |  |
| Actein | 18642-44-9 | Sigma-Aldrich, St. Louis, MO |
| Allocryptopine | 48216-02-0 | Sigma-Aldrich, St. Louis, MO |
| Caffeic acid | 331-39-5 | Sigma-Aldrich, St. Louis, MO |
| Cimicifugoside H-1 | 163046-73-9 | Planta Analytica, Danbury, CT |
| Cimifugin | 37921-38-3 | ChromaDex, Irvine, CA |
| Cimiracemoside C | 256925-92-5 | ChromaDex, Irvine, CA |
| 27-Deoxyactein | 264624-38-6 | ChromaDex, Irvine, CA |
| 26-Deoxycimicifugoside | 214146-75-5 | Carbosynth Ltd., Compton, Berkshire, UK |
| Ferulic Acid | 208-679-7 | ChromaDex, Irvine, CA |
| Formononetin | 485-72-3 | Sigma-Aldrich, St. Louis, MO |
| Isoferulic Acid | 25522-33-2 | Sigma-Aldrich, St. Louis, MO |
| Magnofluorine | 2141-09-5 | Sigma-Aldrich, St. Louis, MO |
| Prim-o-glucosylcimifugin | 80681-45-4 | ChromaDex, Irvine, CA |
| Protocatechuic Acid | 99-50-3 | HWI Analytik GmbH^a^ |
|  |  |  |

^a^Obtained through Sigma-Aldrich, St. Louis, MO
