# Supplementary material for: A cross‐sectional clinical study in women to investigate possible genotoxicity and hematological abnormalities related to the use of black cohosh botanical dietary supplements
Source: Environ Mol Mutagen. 2022 Nov 28;63(8-9):389–99. doi: 10.1002/em.22516 (PMC10018809; doi:10.1002/em.22516)
Supplement: Supplementary file 2 — Table S2 Cohosh materials compared to black cohosh dietary supplements taken by study participants. [file EM-63-389-s002.docx]

**TABLE SII** Cohosh materials compared to black cohosh dietary supplements taken by study participants

|  |  |  |
| --- | --- | --- |
| **Cohosh Material** | **Latin Name** | **Supplier** |
|  |  |  |
|  |  |  |
| Black cohosh root extract^a^ | *Actaea racemosa* L. | PlusPharma, Inc., Vista, CA |
| Black cohosh root XRM^b,c^ | *Actaea racemosa* L. | ChromaDex, Irvine, CA |
| Black cohosh root extract | *Actaea racemosa* L. | U.S. Pharmacopeia, Rockville, MD |
| Chinese *Cimicifuga* root VBRM^d^ | *Actaea dahirica* | ChromaDex, Irvine, CA |
| *Cimicifuga foetida* rhizome granules | a.k.a. *Actaea cimifuga* | Baicao Acu & Moxa, Tampa, FL |
| Red cohosh root VBRM^d^ | *Actaea rubra* | ChromaDex, Irvine, CA |
| Yellow cohosh root VBRM^d^ | *Actaea podocarpa* | ChromaDex, Irvine, CA |
| Remifemin^®^ Menopause Relief Tablets | *Actaea racemosa* L. | Enzymatic Therapy, Inc., Green Bay, WI |
|  |  |  |

^a^Material used for testing conducted by the NTP (Bernacki et al., 2019; Mercado-Feliciano et al., 2012; Smith-Roe et al., 2018)

^b^Extract botanical reference material

^c^Material used for testing conducted by the NTP (“NTP BCE”) (Bernacki et al., 2019; Smith-Roe et al., 2018)

^d^Vouchered botanical reference material
