# Supplementary material for: A cross‐sectional clinical study in women to investigate possible genotoxicity and hematological abnormalities related to the use of black cohosh botanical dietary supplements
Source: Environ Mol Mutagen. 2022 Nov 28;63(8-9):389–99. doi: 10.1002/em.22516 (PMC10018809; doi:10.1002/em.22516)
Supplement: Supplementary file 3 — Table S3 Analysis method parameters for quantitation of black cohosh materials. Coefficients of determination for standards: limit of detection (LOD), lower limit of quantitation (LLOQ), and upper limit of quantitation (ULOQ) [file EM-63-389-s007.docx]

**TABLE SIII** Analysis method parameters for quantitation of black cohosh materials.

Coefficients of Determination for standards: Limit of detection (LOD),

lower limit of quantitation (LLOQ), and upper limit of quantitation (ULOQ)

| **Compound** | **r^2^** | **LOD**  **(μg/ml)** | **Analytical range**  **(ug/ml)** |
| --- | --- | --- | --- |
|  |  |  |  |
| Actein | 0.9788 | 2.55 | 17.2 - 143 |
| Allocryptopine | 0.9915 | 1.70 | 17.1 - 285 |
| Caffeic Acid | 0.9918 | 6.57 | 17.0 - 142 |
| Cimicifugoside H-1 | 0.9889 | 4.44 | 17.5 - 291 |
| Cimifugin | 0.9935 | 6.36 | 17.5 - 146 |
| Cimiracemoside C | 0.9933 | 3.18 | 17.4 - 291 |
| 27-Deoxyactein | 0.9900 | 3.39 | 17.2 - 286 |
| 26-Deoxycimicifugoside | 0.9883 | 3.18 | 17.0 - 284 |
| Ferulic Acid | 0.9893 | 1.27 | 18.1 - 302 |
| Formononetin | 0.9951 | 4.89 | 17.4 - 290 |
| Isoferulic Acid | 0.9976 | 2.33 | 17.7 - 295 |
| Magnoflorine | 0.9878 | 5.31 | 17.8 - 297 |
| Prim-O-Glucosylcimifugin | 0.9914 | NA^a^ | 17.8 - 296 |
| Protocatechuic Acid | 0.9915 | 3.60 | 18.0 - 300 |
|  |  |  |  |

^a^One replicate of the LLOQ was excluded due to poor accuracy and the

LOD could not be calculated.
