# Supplementary material for: A cross‐sectional clinical study in women to investigate possible genotoxicity and hematological abnormalities related to the use of black cohosh botanical dietary supplements
Source: Environ Mol Mutagen. 2022 Nov 28;63(8-9):389–99. doi: 10.1002/em.22516 (PMC10018809; doi:10.1002/em.22516)
Supplement: Supplementary file 4 — Table S4 Hematological endpoints. [file EM-63-389-s004.docx]

**TABLE SIV** Hematological endpoints.

| **Endpoint^a^** | **Group** | **n^b^** | **Mean ± SE** | **25%** | **Median** | **75%** | **p value** |
| --- | --- | --- | --- | --- | --- | --- | --- |
|  |  |  |  |  |  |  |  |
| Hematocrit^c^ | Control | 27 | 39.6 ± 0.6 | 38.7 | 40.1 | 40.9 | 1.000 |
|  | BC Supplement | 23 | 39.5 ± 0.7 | 37.0 | 39.6 | 42.0 |  |
|  |  |  |  |  |  |  |  |
| Red blood cell count^c^ | Control | 27 | 4.35 ± 0.1 | 4.06 | 4.37 | 4.74 | 0.277 |
|  | BC Supplement | 23 | 4.29 ± 0.1 | 4.16 | 4.26 | 4.46 |  |
|  |  |  |  |  |  |  |  |
| White blood cell count^c^ | Control | 27 | 5.78 ± 0.2 | 5.00 | 5.80 | 6.70 | 0.215 |
|  | BC Supplement | 23 | 5.51 ± 0.3 | 4.30 | 5.10 | 6.40 |  |
|  |  |  |  |  |  |  |  |
| Platelet count^c^ | Control | 27 | 250.9 ± 13 | 198.0 | 239.0 | 292.0 | 0.846 |
|  | BC Supplement | 23 | 242.7 ± 8 | 216.0 | 243.0 | 270.0 |  |
|  |  |  |  |  |  |  |  |
| Mean platelet volume (MPV)^c^ | Control | 13 | 9.5 ± 0.3 | 8.7 | 9.6 | 10.3 | 0.922 |
|  | BC Supplement | 5 | 9.7 ± 0.3 | 9.5 | 9.5 | 10.2 |  |
|  |  |  |  |  |  |  |  |
| Hemoglobin^c^ | Control | 27 | 12.7 ± 0.2 | 12.2 | 13.0 | 13.2 | 0.635 |
|  | BC Supplement | 23 | 12.9 ± 0.2 | 11.8 | 13.1 | 13.8 |  |
|  |  |  |  |  |  |  |  |
| Mean corpuscular volume (MCV)^c^ | Control | 27 | 91.6 ± 1.2 | 86.9 | 90.7 | 96.3 | 0.457 |
|  | BC Supplement | 23 | 92.3 ± 1.2 | 89.6 | 92.8 | 96.4 |  |
|  |  |  |  |  |  |  |  |
| Mean corpuscular hemoglobin (MCH)^c^ | Control | 27 | 29.4 ± 0.4 | 28.0 | 29.1 | 30.7 | 0.078 |
|  | BC Supplement | 23 | 30.1 ± 0.5 | 29.5 | 30.4 | 31.2 |  |
|  |  |  |  |  |  |  |  |
| Mean corpuscular hemoglobin conc. (MCHC)^c^ | Control | 27 | 32.1 ± 0.2 | 31.8 | 32.3 | 32.7 | 0.316 |
|  | BC Supplement | 23 | 32.6 ± 0.3 | 31.7 | 32.4 | 33.5 |  |
|  |  |  |  |  |  |  |  |
| Red cell distribution width (RDW)^c^ | Control | 27 | 15.2 ± 0.3 | 14.0 | 14.7 | 15.9 | 0.008^e^ |
|  | BC Supplement | 23 | 14.0 ± 0.3 | 13.0 | 13.6 | 14.7 |  |
|  |  |  |  |  |  |  |  |
| Reticulocyte (absolute)^d^ | Control | 26 | 69.1 ± 3.9 | 58.1 | 66.8 | 82.5 | 0.765 |
|  | BC Supplement | 22 | 65.5 ± 3.7 | 48.3 | 67.0 | 80.8 |  |
|  |  |  |  |  |  |  |  |
| Reticulocyte (%)^d^ | Control | 26 | 1.68 ± 0.2 | 1.32 | 1.59 | 1.90 | 0.742 |
|  | BC Supplement | 22 | 1.54 ± 0.1 | 1.19 | 1.62 | 1.76 |  |
|  |  |  |  |  |  |  |  |
| Reticulocyte (hemoglobin)^d^ | Control | 26 | 34.1 ± 0.6 | 32.9 | 33.9 | 36.3 | 0.485 |
|  | BC Supplement | 22 | 34.4 ± 0.6 | 33.1 | 34.8 | 36.3 |  |
|  |  |  |  |  |  |  |  |
|  |  |  |  |  |  |  |  |
| Immature reticulocyte fraction^d^ | Control | 26 | 8.81 ± 0.8 | 5.4 | 7.9 | 11.0 | 0.109 |
|  | BC Supplement | 22 | 6.67 ± 0.6 | 4.5 | 6.1 | 9.3 |  |
|  |  |  |  |  |  |  |  |
| Immature platelet fraction^d^ | Control | 26 | 5.01 ± 0.7 | 2.8 | 4.1 | 6.9 | 0.992 |
|  | BC Supplement | 21 | 4.90 ± 0.6 | 3.5 | 4.1 | 5.5 |  |
|  |  |  |  |  |  |  |  |

^a^Several endpoints were not analyzed due to extremely low sample sizes (n = 2) in the BC supplement group: neutrophils, lymphocytes,

monocytes, eosinophils, basophils, and the absolute counts for each of these cell types.

**^b^**Number of participants in each group for which data were available for each endpoint that was measured.

^c^Analyzed at Quest Diagnostics.

^d^Analyzed at NIH Clinical Center.

^e^Control and BC Supplement group differ significantly at p < 0.05.
