# Supplementary material for: A cross‐sectional clinical study in women to investigate possible genotoxicity and hematological abnormalities related to the use of black cohosh botanical dietary supplements
Source: Environ Mol Mutagen. 2022 Nov 28;63(8-9):389–99. doi: 10.1002/em.22516 (PMC10018809; doi:10.1002/em.22516)
Supplement: Supplementary file 5 — Table S5 Participant micronucleus values. [file EM-63-389-s001.docx]

**Table SV** Participant micronucleus values.

|  |  | | | | | | |
| --- | --- | --- | --- | --- | --- | --- | --- |
|  | **Control group** | | | | **Supplement group** | | |
|  | **%MN-RET** | | **%RET** | | **%MN-RET** | | **%RET** |
|  | 0.03 | | 0.135 | | 0.06 | | 0.259 |
|  | 0.07 | | 0.183 | | 0.07 | | 0.127 |
|  | 0.09 | | 0.142 | | 0.13 | | 0.037 |
|  | 0.11 | | 0.244 | | 0.14 | | 0.123 |
|  | 0.14 | | 0.390 | | 0.16 | | 0.132 |
|  | 0.14 | | 0.100 | | 0.17 | | 0.022 |
|  | 0.15 | | 0.168 | | 0.18 | | 0.068 |
|  | 0.15 | | 0.377 | | 0.19 | | 0.133 |
|  | 0.16 | | 0.308 | | 0.20 | | 0.083 |
|  | 0.17 | | 0.087 | | 0.21 | | 0.103 |
|  | 0.17 | | 0.103 | | 0.21 | | 0.106 |
|  | 0.19 | | 0.050 | | 0.23 | | 0.191 |
|  | 0.19 | | 0.097 | | 0.23 | | 0.021 |
|  | 0.20 | | 0.210 | | 0.24 | | 0.139 |
|  | 0.21 | | 0.085 | | 0.25 | | 0.065 |
|  | 0.22 | | 0.141 | | 0.26 | | 0.136 |
|  | 0.23 | | 0.046 | | 0.31 | | 0.051 |
|  | 0.28 | | 0.053 | | 0.34 | | 0.047 |
|  | 0.29 | | 0.062 | | 0.39 | | 0.118 |
|  | 0.34 | | 0.063 | | 0.47 | | 0.040 |
|  | 0.73 | | 0.029 | | 0.60 | | 0.019 |
|  | 0.76 | | 0.091 | | 0.75 | | 0.015 |
|  |  | |  | | 0.89 | | 0.033 |
|  |  | |  | |  | |  |
| Mean ± SE | 0.228 ± 0.04 | | 0.144 ± 0.02 | | 0.290 ± 0.04 | | 0.090 ± 0.01 |
|  |  |  | |  | |  | |

^a^ %MN-RET, percent micronucleated reticulocytes. Previously published (Torous et al., 2020)

mean value for 338 males and females ranging in age from birth (cord blood) to 73 years, 0.15 ± 0.10%;

range, 0.01 – 0.79%.

^b^ %RET, percentage of reticulocytes among total circulating erythrocytes. Previously published

range was generally 0.1 – 0.3% (Torous et al., 2020).
