# Supplementary figures and images for: A cross‐sectional clinical study in women to investigate possible genotoxicity and hematological abnormalities related to the use of black cohosh botanical dietary supplements
Source: Environ Mol Mutagen. 2022 Nov 28;63(8-9):389–99. doi: 10.1002/em.22516 (PMC10018809; doi:10.1002/em.22516)

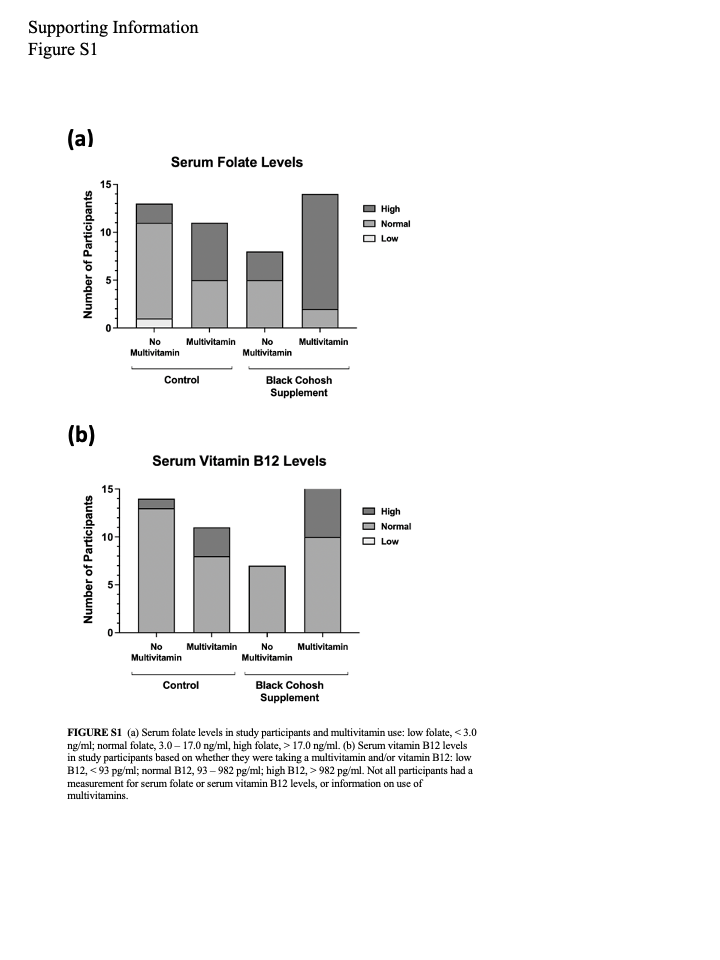

Supplement: Supplementary file 6 — Figure S1 (a) Serum folate levels in study participants and multivitamin use: Low folate, < 3.0 ng/ml; normal folate, 3.0–17.0 ng/ml, high folate, > 17.0 ng/ml. (b) Serum vitamin B12 levels in study participants based on whether they were taking a multivitamin and/or vitamin B12: low B12, < 93 pg/ml; normal B12, 93–982 pg/ml; high B12, > 982 pg/ml. Not all participants had a measurement for serum folate or serum vitamin B12 levels, or information on use of multivitamins. [file EM-63-389-s006.tiff]
